# Use of anthropophilic culicid-based xenosurveillance as a proxy for *Plasmodium vivax* malaria burden and transmission hotspots identification

**DOI:** 10.1371/journal.pntd.0006909

**Published:** 2018-11-12

**Authors:** Joabi Nascimento, Vanderson S. Sampaio, Stephan Karl, Andrea Kuehn, Anne Almeida, Sheila Vitor-Silva, Gisely Cardoso de Melo, Djane C. Baia da Silva, Stefanie C. P. Lopes, Nelson F. Fé, José B. Pereira Lima, Maria G. Barbosa Guerra, Paulo F. P. Pimenta, Quique Bassat, Ivo Mueller, Marcus V. G. Lacerda, Wuelton M. Monteiro

**Affiliations:** 1 Diretoria de Ensino e Pesquisa, Fundação de Medicina Tropical Doutor Heitor Vieira Dourado, Manaus, AM, Brazil; 2 Escola Superior de Ciências da Saúde, Universidade do Estado do Amazonas, Manaus, AM, Brazil; 3 Population Health & Immunity Division, Walter & Eliza Hall Institute, Parkville, Australia; 4 Entomology Section, Vector-borne Diseases Unit, Papua New Guinea Institute of Medical Research, Papua, New Guinea; 5 Department of Medical Biology, University of Melbourne, Australia; 6 Instituto Leônidas & Maria Deane, Fundação Oswaldo Cruz, Manaus, AM, Brazil; 7 Laboratório de Fisiologia e Controle de Artrópodes Vetores, Instituto Oswaldo Cruz (Fiocruz), Rio de Janeiro, RJ, Brazil; 8 Laboratório de Entomologia Médica, Centro de Pesquisas René Rachou (Fiocruz), Belo Horizonte, MG, Brazil; 9 ISGlobal, Hospital Clínic—Universitat de Barcelona, Barcelona, Spain; 10 Centro de Investigação em Saúde de Manhiça (CISM), Maputo, Mozambique; 11 ICREA, Barcelona, Spain; 12 Pediatric Infectious Diseases Unit, Pediatrics Department, Hospital Sant Joan de Déu (University of Barcelona), Barcelona, Spain; 13 Parasites & Hosts Unit, Institut Pasteur, Paris, France; Centers for Disease Control and Prevention, Puerto Rico, UNITED STATES

## Abstract

Vector-borne diseases account for more than 17% of all infectious diseases, causing more than one million deaths annually. Malaria remains one of the most important public health problems worldwide. These vectors are bloodsucking insects, which can transmit disease-producing microorganisms during a blood meal. The contact of culicids with human populations living in malaria-endemic areas suggests that the identification of *Plasmodium* genetic material in the blood present in the gut of these mosquitoes may be possible. The process of assessing the blood meal for the presence of pathogens is termed ‘xenosurveillance’. In view of this, the present work investigated the relationship between the frequency with which *Plasmodium* DNA is found in culicids and the frequency with which individuals are found to be carrying malaria parasites. A cross-sectional study was performed in a peri-urban area of Manaus, in the Western Brazilian Amazon, by simultaneously collecting human blood samples and trapping culicids from households. A total of 875 individuals were included in the study and a total of 13,374mosquito specimens were captured. Malaria prevalence in the study area was 7.7%. The frequency of households with at least one culicid specimen carrying *Plasmodium* DNA was 6.4%. *Plasmodium* infection incidence was significantly related to whether any *Plasmodium* positive blood-fed culicid was found in the same household [IRR 3.49 (CI95% 1.38–8.84); p = 0.008] and for indoor-collected culicids [IRR 4.07 (CI95%1.25–13.24); p = 0.020]. Furthermore, the number of infected people in the house at the time of mosquito collection was related to whether there were any positive blood-fed culicid mosquitoes in that household for collection methods combined [IRR 4.48 (CI95%2.22–9.05); p<0.001] or only for indoor-collected culicids [IRR 4.88 (CI95%2.01–11.82); p<0.001]. Our results suggest that xenosurveillance can be used in endemic tropical regions in order to estimate the malaria burden and identify transmission foci in areas where *Plasmodium vivax* is predominant.

## Author summary

In the era of malaria elimination, novel surveillance strategies are needed in order to achieve and sustain malaria free status in the region. New malaria infection burden surveillance strategies should be simple to implement, technologically uncomplicated, cost-effective and applicable to areas where malaria cases are known to occur. In this context, the xenosurveillance is an important tool, especially when using widely distributed and anthropophilic mosquitoes. *Culex* mosquitoes do not transmit malaria, but due to their wide dispersion and anthropophilic behavior, may be used for the monitoring of certain vector-borne diseases, such as malaria, whose agent can be easily identified through molecular techniques in the ingested blood. Here, we evaluated a *Plasmodium* xenosurveillance approach to determine human malaria prevalence in three distinct peri-urban areas of Manaus. For this purpose, we evaluated the presence of *Plasmodium* DNA in engorged culicids sampled indoors regardless of their role in malaria transmission and compared them with the human infection status in the same households. The spatial correlation between the presence of Plasmodium DNA in mosquitoes was similar to the infection rate in humans which clearly indicates that xenosurveillance can be a complimentary strategy in endemic tropical regions in order to estimate the malaria burden.

## Introduction

Vector-borne diseases account for more than 17% of all infectious diseases, and cause more than one million deaths annually. Vectors are living organisms that can transmit infectious diseases between humans or from animals to humans. These vectors are bloodsucking insects, which ingest disease-producing microorganisms during a blood meal from an infected host (human or animal) and can subsequently inject them into a new host during a new blood meal. Mosquitoes are among the best-known disease vectors. Three genera are responsible for transmitting a series of life-threatening diseases worldwide, however mostly in tropical areas: 1) *Aedes*: vector of Chikungunya, dengue, Zika, Rift Valley and yellow fever; 2) *Anopheles*: vector of malaria, Saint Louis encephalitis, West Nile fever and several *Anopheles* A and B orthobunyaviruses and 3) *Culex*: Japanese encephalitis, lymphatic filariasis, Saint Louis encephalitis, Oropouche and West Nile fever [1,2].

Many of these diseases are preventable through informed, protective measures. Surveillance is critical for the prediction of future disease outbreaks and epidemics at an early stage, as well as for identifying transmission hotspots. Among vector-borne diseases, malaria remains one of the most important public health problems worldwide. It is estimated that malaria transmission still occurs in 91 countries and territories of the world, and causes an estimated 216 million clinical episodes and around 445,000 deaths globally every year [3]. An unknown, and much higher, number of individuals in malaria-endemic areas silently carry malaria parasites, which provides a reservoir for malaria transmission. The challenge of identifying them has been recognized as a major difficulty in regard to the adequate control and eventual elimination of malaria [4].

The majority of mosquito species is hematophagous, and relies on blood from vertebrates for nourishment and reproduction. When engorged, mobility is limited and they can be easily collected via aspiration. Blood meal analysis is then carried out in order to detect the presence of pathogens, a process termed ‘xenosurveillance’ [5,6]. Experimentally, mosquitoes can be used as biological syringes’ so we can accurately quantify the presence of viruses or other microorganisms in their midgut and also evaluate the potential role of small vertebrates in the transmission cycle of arboviruses [7]. Such methods have shown, for instance, transmission of human pathogens, such as hepatitis B virus (HBV) [8,9] and dengue virus (DENV) by *Culex quinquefasciatus* [10] and hepatitis C virus (HCV) by *Culex pipiens* complex [11,12]. Field studies have confirmed that vertebrate viral pathogens that are not vector-borne could also be detected in the blood meals of mosquitoes belonging to a variety of taxa [13,14]. H5N1 virus sequences were found in blood-engorged mosquitoes collected near a poultry farm during an outbreak of avian influenza in Thailand [15]. To survey DENV and Japanese encephalitis circulation in this same country, an enzyme-linked immunosorbent assay detected virus-reactive antibodies in blood meals collected from culicids irrespective of the mosquito species’ ability as a vector [16]. Nucleic acid sequences from human papillomavirus 23 (HPV23), human herpesvirus 1 and human papillomavirus type 112 (HPV112) were identified in mosquito samples from San Diego, California [17]. In Central Brazil, DENV-4 was detected in several culicids, especially *Cx*. *quinquefasciatus* [18]. In Liberia, *An*. *gambiae* bloodfed mosquitoes resting indoors were found positive for human skin-associated microbes (e.g. *Staphylococcus epidermidis* and *Propionibacterium acnes*), Epstein-Barr virus (EBV) and canine distemper virus (CDV) signatures [6]. Human bacterial pathogens such as *Rickettsia* spp. [19,20], *Francisella tularensis* [21] and *Borrelia* [22,23] were also found in field-collected culicids.

In the case of malaria, clinical, parasitological and serological markers of transmission, as detected in humans, have been routinely and historically used to detect transmission pockets and guide hotspot-targeted interventions. However, in more recent times, interest has also shifted to evaluating transmission through the exploration of the burden of low-density (formerly described as “asymptomatic” albeit now recognized to be potential causes of more silent, but still important, clinical consequences) infections in low transmission areas [24–27]. Nevertheless, current malaria surveillance systems can be expensive, laborious and useless for sampling numbers of exposed individuals over space and time. In Liberia, a pathogen surveillance strategy using xenosurveillance has found the presence of *P*. *falciparum* in *An*. *gambiae*; however, this result was expected since malaria is holoendemic in this region and therefore the mosquito or/and the human upon which it fed may be infected [6].

Fauver et al. [9] have demonstrated the viability of xenosurveillance as a tool for sampling human blood in order to detect circulating pathogens. However, to the best of our knowledge, xenosurveillance of malaria using highly anthropophilic culicids, frequently more abundant than anophelines, has been never validated in regards to its fundamental utility, feasibility in field timeframes and technically relevant concentrations. In this work, we hypothesized that, in this surveillance approach, molecular detection of *Plasmodium* in blood fed mosquitoes reflect the malaria burden and make malaria surveillance more feasible in tropical localities where *Culex* is abundant.

## Methods

### Study site

This study was conducted in the Brasileirinho, Puraquequara and Ipiranga communities, in a peri-urban area of Manaus, Western Brazilian Amazon. According to a census carried out by the field team before the beginning of the study, approximately 2,400 inhabitants were living in the study area, of around 72 km^2^. The general characteristics of these communities were previously described [28,29].

### Malaria infection status in humans

A cross-sectional sampling was performed from January to March, 2014by simultaneously collecting human blood samples and trapping culicids from 233 households located in this area. Sampling included all inhabitants living in these houses that were willing to participate in the study. For each study participant, a questionnaire was completed, containing personal malaria preventive measure information and the participants’ history of malaria episodes in the preceding 30 days (Table 1). Upon enrolment, a 300 μL blood sample was collected from the participant via finger puncture using Microtainer tubes containing EDTA and sodium fluoride (Becton Dickinson, NJ, USA). In infants, blood was obtained by puncture of the heel or toe. All samples were frozen at -8°C until further processing.

**Table 1:** Characteristics of the households sampled for mosquito collections.

Variable	Number	%
**Community**		
Ipiranga	103	44.2
Brasileirinho	81	34.8
Puraquequara	49	21.0
**Location**		
Main road	84	36.1
Side road	149	63.9
**Number of residents**		
1	83	31.8
2	64	24.5
3	37	14.2
4	25	9.6
≥5	52	19.9
**House building type**		
Brick	133	57.1
Wood	100	42.9
**Walls**		
Complete	221	94.9
Partial	12	5.1
**Cracks in the walls**		
No	175	75.1
Yes	58	24.9
**Doors and windows**		
No	3	1.3
Yes	230	98.7
**Long-lasting insecticidal nets (LLINs)**		
No	181	77.7
Yes	52	22.3
**Indoor residual spraying (IRS) in the last 6 months**		
No	126	54.1
Yes	107	45.9
**Insecticide-treated bednet use during the previous last night**^**1**^		
No	95	40.8
Yes	138	59.2
**Fever**^**1**^^,^^**2**^		
No	219	94.0
Yes	14	6.0
**Use of antimalarial drugs in the last 30 days**^**2**^		
No	203	87.1
Yes	30	12.9

^1^at least one resident presenting the variable

^2^body temperature above 37.5°C at the time of blood collection or in the past 48 hours.

In the case of symptoms related to malaria, a thick blood smear was prepared following the World Health Organization guidelines [30]. All participants that tested positive for *P*. *falciparum* and/or *P*. *vivax* by thick blood smear and/or qPCR were considered as infected by malaria parasites, and received treatment according to the guidelines of the Brazilian Ministry of Health [31].

### Detection of *Plasmodium* spp.in human samples

Pelleted RBCs obtained from participants’ blood were resuspended in PBS and genomic DNA was extracted using a FavorPrep 96-well Genomic DNA Kit (Favorgen, Ping-Tung, Taiwan) according to the manufacturer’s instructions. DNA was eluted in elution buffer and stored at -20°C or vacuum concentrated (Concentrator 5301, Eppendorf, Hamburg, Germany) [32].

All DNA samples were subjected to QMAL Taqman qPCR in order to detect any *Plasmodium* spp. by targeting a conserved region of the 18S rRNA gene [30]. QMAL-positive samples were further analysed by Taqman qPCR assays to detect species-specific sequences of 18S rRNA gene of *P*. *falciparum* and *P*. *vivax*, as previously described [32,33]. PCR assays were carried out on the 7500 Fast Real-Time PCR System (Applied Biosystems, Foster City, USA).

### Culicidtrapping and identification

In each household, mosquitos were collected using BG-Sentinel traps with BG-Lure attractant and electric entomological aspirators (Horst Armadilhas Ltda., Brazil). One BG-Sentinel per house was left generally in a bedroom for around 24 hours. Aspiration inside the dwellings lasted approximately 5 to 15 minutes, depending on the number and size of the rooms. In the collection period, average temperature was 27.4°C (23.4°C- 30.8°C). The captured culicids were transferred by suitable plastic cages to the FMT-HVD Entomology Laboratory on the same day. The mosquitoes were placed on petri dishes arranged on ice, immediately identified in a dormant state and separated by sex and blood-feeding status (engorged or not). Taxonomical identification was made using the keys of Consoli and Lourenço-de-Oliveira [34], and Faran and Linthicum [35]. After the identification, engorged female mosquitos were stored individually in ethanol 80% v/v at -20°C until DNA extraction.

### Standardization of *Plasmodium* DNA detection from engorged culicids

The number of mosquitoes per pool and the maximum post-feeding time in which *Plasmodium* DNA detection is still possible were determined by using experimentally fed culicids. A colony of *Cx*. *quinquefasciatus* was established from immature forms (larvae and pupae) of the mosquitoes collected in natural breeding environments on the outskirts of the city. *Cx*. *quinquefasciatus*, the most abundant mosquito species found on the outskirts of Manaus [36], was chosen for this purpose. These were maintained under standard insectary conditions of 27°C, 80% relative humidity, and a 12:12 light/dark cycle until we obtained adult F1 generation, according to Gerberg et al. methodology [37]. 100 to 120 5-day-old female mosquitoes were given a blood meal with *P*. *vivax* obtained from a volunteer’s blood, using an artificial membrane feeding system [38]. After feeding, 50 fully engorged females were immediately sacrificed by freezing at -20°C. The remaining fully engorged females were returned to insectary conditions, and subsequent samples of 10 mosquitoes were sacrificed daily from day 1 (D1) to 10 (D10) after a *P*. *vivax* blood meal. In order to estimate the maximum number of mosquitos per pool for *Plasmodium* sp. detection, different proportions of infected blood fed per non-infected blood fed mosquitos were assessed: 1:1; 1:3; 1:5; 1:7; and 1:9. For this assay, only abdomens were used for DNA extraction and QMAL q-PCR.

### DNA extraction from mosquitos

DNA extraction was performed according to Musapa et al. [39] by using a 5% w/v Chelex 100 Resin (styrene divinylbenzene copolymer containing paired iminodiacetate ions; Bio-Rad, Hercules, CA, USA). All DNA samples were subjected to QMAL Taqman qPCR to detect *Plasmodium* spp. by targeting a conserved region of the 18S rRNA gene [32,33].

### Data analysis

The design of the standardized forms, and their scanning, processing and exporting to Excel sheets was performed using Cardiff Teleform v. 10.4.1 (Cardiff Software). Statistical analysis was performed using Stata 13.1 and QGis v. 2.18.7. Since houses were used as unit of analysis, information on building conditions, spatial coordinates, demography and infection status in both human and mosquitos were clustered by household.

Relative frequency of *Plasmodium* DNA presence in culicids was determined by the number of positive pools with QMAL q-PCR genus-specific amplification per total number of pools from the same household. Similarly, the human infection rate was calculated as the proportion of QMAL q-PCR genus-specific positive per total of samples assessed for each household. Although the number of copies could be obtained from both tests, the data was dichotomized as positive or negative for analysis purposes. Negative binomial regression was used to assess association between presence of any positive blood-fed *Culex* in a household and two distinct variables: (i) the total number of new infections per time at risk (incidence); and (ii) number of people infected at the time of mosquito collection in the household (i.e., household prevalence). Additionally, the same models were carried out using the total number of blood-fed *Culex* mosquitoes as independent variable. Differences were considered statistically significant for p<0.05.

### Ethics statement

Colonised *Cx*. *quinquefasciatus* blood feeding on *Mus musculus* was approved by the FMT-HVD Ethics Committee in Animal Research (349.211/2013). The human survey was approved by the Brazilian National Committee of Ethics (349.211/2013). An informed consent form was signed by all study participants or by a parent or legal guardian. Children between 12 and 17 years signed an additional assent form. Malaria patients were treated according the Brazilian Ministry of Health guidelines [31].

## Results

### Individual and household characteristics

A total of 875 individuals were included in the study, and nearly half of them (45.6%) were 0 to 20 years old and 50.7% were women. A total of 381 (43.5%) individuals reported to use long-lasting insecticidal nets in the preceding night and 556 (63.5%) reported that their houses had been sprayed with permethrin in the preceding six months. A total of 244 (27.9%) individuals reported no previous malaria episode, 252 (28.8%) reported 1 to 3 previous episodes and 379 (43.3%) reported having experienced more than 3 episodes in their lifetime. Malaria prevalence in the study area was 7.7%, ranging from 3.2% in the Brasileirinho community to 11.2% in the Puraquequara community. In the Ipiranga community, prevalence was 8.1%. Only 3 (4.1%) of the total 61 detected infections were symptomatic.

From the total of households included, 44.2% were located in the Ipiranga community, 34.76% in the Brasileirinho community and 21.03% in the Puraquequara community. The mean number of residents per household was 3, but ranged anywhere from 1 to 15. Regarding the household structure, bricks were mostly used for building (133; 57.1%), houses with complete walls (221; 94.9%). 58 (24.9%) of the houses presented cracks in the walls. 52 (22.3%) houses possessed long-lasting insecticidal nets, 107 (45.9%) had been sprayed in the last 6 months and 138 (59.2%) had at least one resident who had used long-lasting insecticide-treated nets during the previous night. Fever was documented in at least one resident in 14 (6%) of the households. Thirty (12.9%) households had at least one resident who had been using antimalarials in the last 30 days (Table 1).

### Standardization of *Plasmodium* DNA detection from engorged culicids

**Fig 1:**
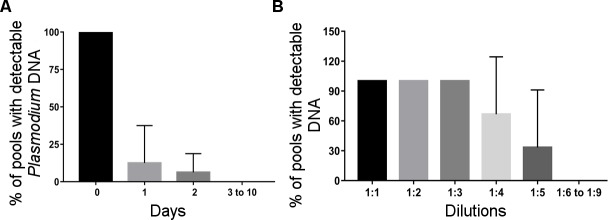
Standardization of *Plasmodium* DNA detection from engorged culicids. *Plasmodium* DNA was detectable at D2 post-feeding (A) and 1:5 mosquito pool size (B).

In the search for the definition of which field collection mosquitoes should be used for the genus-specific qPCR assays, experimental infections were performed using *Cx*. *quinquefasciatus* from a pre-established colony. *Plasmodium* DNA was detectable at D2 post-feeding, when the *Plasmodium* DNA carriage was reduced to 12.5%. For this, we chose to test only visibly blood-fed mosquitoes. Detection using qPCR was possible up to a pool size of 5 mosquitos (Fig 1).

### Culicids abundance and *Plasmodium* DNA positivity

A total of 13,374 specimens were captured: 12,132 (90.7%) by using BG-Sentinel traps and 1,242 (9.3%) b electric aspirators. Most of the mosquitoes (n = 12,923; 96.6%) were *Culex* spp. with *Cx*. *quinquefasciatus* being the most common species (n = 12,599; 94.2%). 293 of the culicids (2.2%) were not identified. The mean number of culicids per household was 50, and ranged from 0 to 887 specimens; in 76.6% of the households 0–50 culicids were collected, and 6.1% presented more than 150 culicids. BG-Sentinel traps (12,132; 90.7%) were more effective in capturing mosquitoes and culicid diversity was greater (at least 10 taxa) when compared to electric aspiration (at least 6 taxa). *Cx*. *quinquefasciatus* predominated in the BG-Sentinel traps (95.3%) and the electric aspiration (83.6%) collections.

A proportion of 17.7% of the culicids were engorged, ranged from 14.5% in BG-Sentinel trapped specimens to 48.1% in those collected by electric aspirations (Chi-square 875.09, p<0.0001). For *Cx*. *quinquefasciatus*, 16.2% of the culicids were engorged, with 13.8% of BG-Sentinel trapped specimens and 42.0% of culicids from aspirations (Chi-square 557.08, p<0.001) (Table 2). The mean number of engorged culicids per household was 7.7, ranging from 0 to 176 specimen. A total of 198 households (75.9%) possessed at least one engorged mosquito in the collected specimens. The majority of the households possessed 1–5 engorged mosquitoes (121; 46.4%).

**Table 2:** Frequency of culicids collected according to trapping method.

Culicid taxon	Electric aspirators		BG-Sentinel traps		Total	
	Number	Blood-fed specimens (%)	Number	Blood-fed specimens (%)	Number	Blood-fed specimens (%)
*Culex quinquefasciatus*	1,038	436 (42.0)	11,561	1,601 (13.8)	12,599	2,037 (16.2)
*Culex* sp.	98	82 (83.7)	225	77 (34.2)	323	159 (49.2)
Unidentified culicids	89	71 (79.8)	204	70 (34.3)	293	141 (48.1)
*Anopheles darlingi*	12	6 (50.0)	122	16 (13.1)	134	22 (16.4)
*Anopheles* sp.	1	0 (0)	6	0 (0)	7	0 (0)
*Psorophora* sp.	2	2 (100.0)	2	0 (0)	4	2 (50.0)
*Uranotaenia* sp.	0	. . .	4	0 (0)	4	0 (0)
*Limatus* sp.	0	. . .	3	0 (0)	3	0 (0)
*Aedeomyia* sp.	0	. . .	2	0 (0)	2	0 (0)
*Coquillettidia* sp.	0	. . .	1	0 (0)	1	0 (0)
*Culex* (*Anoedioporpa*)	0	. . .	1	0 (0)	1	0 (0)
*Culexspisseps*	1	1 (100.0)	0	. . .	1	1 (100.0)
*Psorophora albigenu*	1	0 (0)	0	. . .	1	0 (0)
Toxorhynchitinae	0	. . .	1	0 (0)	1	0 (0)
**Total**	**1,242**	**598 (48.1)**	**12,132**	**1,764 (14.5)**	**13,374**	**2,362 (17.7)**

Two household parameters affected the total number of mosquitoes captured indoors (Table 3): wooden houses (IRR 1.44, CI95%1.04–2.01; p = 0.029) and houses with cracks in the walls (IRR 1.38, CI95% 0.98–1.95; p = 0.067). Houses located in the Ipiranga community generally yielded less mosquitoes in comparison to houses located in the Puraquequara community (IRR 0.64, CI95% 0.44–0.93; p = 0.020). The differences were insignificant when only engorged culicids were analysed. However, the number of engorged mosquitoes was moderately associated with the number of total mosquitoes per house (p<0.0001, R^2^ = 0.37).

**Table 3:** Association of house parameters with total number of mosquitoes and number of blood-fed mosquitoes captured during indoor collections (negative binomial model).

Total culicid number	Total culicid number		Blood-fed culicid number	
	IRR (CI 95%)	P	IRR (CI 95%)	p
**House building type**				
Brick	1		1	
Wood	1.44 (1.04–2.01)	0.029	0.98 (0.63–1.54)	0.938
**Cracks in the walls**	1		1	
Yes	1.38 (0.98–1.95)	0.067	0.98 (0.61–1.59)	0.954
**Community**				
Ipiranga	1		1	
Brasileirinho	0.82 (0.57–1.18)	0.286	0.79 (0.48–1.32)	0.372
Puraquequara	0.64 (0.44–0.93)	0.020	0.63 (0.38–1.05)	0.074

Overall *Plasmodium* DNA positivity in engorged culicids was 2.9%, specifically, 3.2% in mosquitoes collected by electric aspiration and 2.7% collected by BG-Sentinel traps (Chi-square 0.1703, p = 0.679). *Cx*. *quinquefasciatus* showed a positivity rate of 3.4%, with no difference between collection methods (3.6% and 3.3%, respectively; Chi-square 0.0292, p = 0.864). Other *Culex* genus representatives (*Culex* sp.) showed a positivity rate of 3.7%, with no differences between collection methods (5.9% and 2.1%, respectively; Chi-square 0.8149, p = 0.367). *Anopheles darlingi* was found with *Plasmodium* DNA only in BG-Sentinel traps (1.9%). Other culicids did not present *Plasmodium* DNA positive samples (Table 4).

**Table 4:** *Plasmodium* DNA positivity in blood-engorged culicid pools according to species.

Culicid taxon	Electric aspirators		BG-Sentinel traps		Total	
	Number of pools	*Plasmodium* DNA positivity (%)	Number of pools	*Plasmodium* DNA positivity (%)	Number of pools	*Plasmodium* DNA positivity (%)
*Culex quinquefasciatus*	169	3.6	428	3.3	597	3.4
*Culex* sp.	34	5.9	48	2.1	82	3.7
Unidentified culicids	28	0.0	53	0.0	81	0.0
*Anopheles darlingi*	12	0.0	53	1.9	65	1.5
*Anopheles* sp.	1	0.0	6	0.0	7	0.0
*Psorophora* sp.	1	0.0	0	. . .	1	0.0
***Culexspisseps***	**1**	**0.0**	**0**	. . .	**1**	**0.0**
*Psorophora albigenu*	1	0.0	0	. . .	1	0.0
Toxorhynchitinae	0	. . .	1	0.0	1	0.0
**Total**	**247**	**3.2**	**589**	**2.7**	**836**	**2.9**

**Fig 2:**
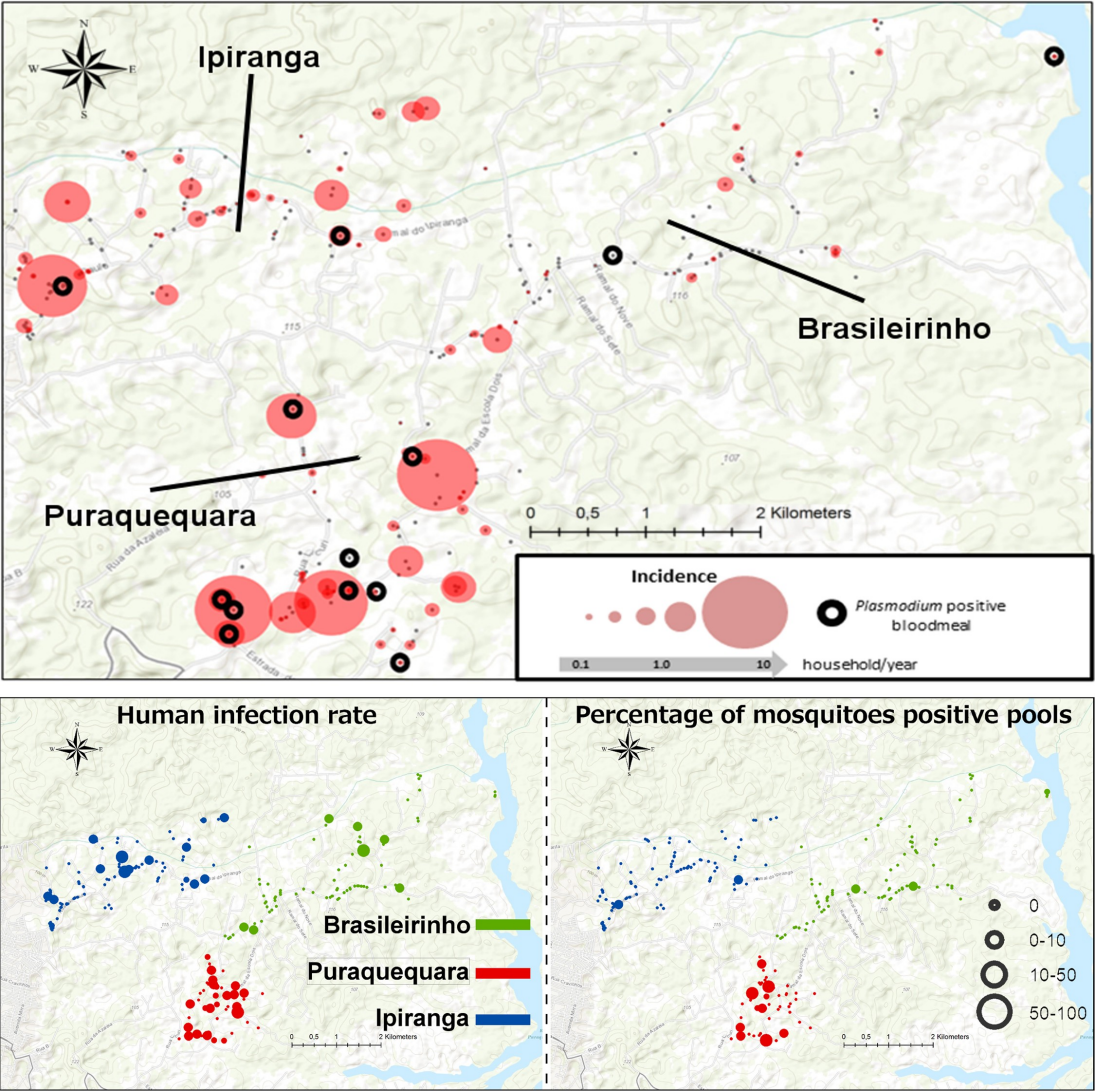
Spatial representation of human *Plasmodium* infection rate and proportion of *Plasmodium* DNA positive pools in the study area. This study was conducted in the Brasileirinho, Puraquequara and Ipiranga communities, in a peri-urban area of Manaus, Western Brazilian Amazon. Culicid xenosurveillance was good in detecting malaria clusters in the study site, and demonstrated coinciding hotspots. This figure was created with Open Street Maps, which are licensed under ODBL 1.0.

The frequency of households with at least one culicid specimen carrying *Plasmodium* DNA was 6.4%. Fig 2 indicates that culicids xenosurveillance was efficient in detecting malaria clusters at the study site. Out of the 14 households with at least one positive culicid, 8 (57%) showed an incidence>0 in humans and 31% had a *Plasmodium* infection incidence >0.

### Predicting malaria burden through the detection of *Plasmodium* DNA in *Culex* mosquitoes

*Plasmodium* infection incidence (as expressed in total number of new infections per household/time at risk) was significantly related to whether any *Plasmodium* positive blood-fed culicids were found in the same house when both collection methods were combined [IRR 3.49 (CI95% 1.38–8.84); p = 0.008] and for indoor-collected culicids [IRR 4.07 (CI95%1.25–13.24); p = 0.020] (Table 5).

**Table 5:** Association of blood-fed and *Plasmodium* DNA positive culicids and force of infection and prevalence of malaria in the study area.

Number of culicids	Total culicids		Outdoor culicids		Indoor culicids	
	IRR (CI 95%)	P	IRR (CI 95%)	p	IRR (CI 95%)	p
***Plasmodium* DNA positive culicids**						
Incidence	3.49 (1.38–8.84)	0.008	4.97 (0.43–57.67)	0.200	4.07 (1.25–13.24)	0.020
Prevalence	4.48 (2.22–9.05)	<0.001	. . .	. . .	4.88 (2.01–11.82)	<0.001
**Blood-fed culicids**						
Incidence	1.03 (1.01–1.05)	0.006	1.05 (0.99–1.11)	0.060	1.01 (0.98–1.04)	0.610
Prevalence	1.01 (0.99–1.03)	0.220	1.04 (0.98–1.09)	0.174	1.01 (0.98–1.04)	0.394

Furthermore, the number of people infected in the house at the time of mosquito collection was related to whether there were any positive blood-fed culicid mosquitoes in that house when both collection methods were combined [IRR 4.48 (CI95%2.22–9.05); p<0.001] or only for indoor-collected culicids [IRR 4.88 (CI95%2.01–11.82); p<0.001].

The total number of blood-fed culicids was also significantly associated with *Plasmodium* incidence (as expressed in total number of new infections per household/time at risk [1.03 (CI95% 1.01–1.05); p = 0.006].

## Discussion

A large majority of mosquito species are hematophagous and rely on vertebrate blood for nourishment and reproduction. The contact of several anthropophilic and endophagic culicids with human populations living in malaria endemic areas suggests that the identification of *Plasmodium* genetic material in the blood present in the gut of mosquitoes may be possible and correlated to infection prevalence in the human population. In view of this, the present study investigated the relationship between *Plasmodium* DNA presence in engorged culicids and the frequency of individuals carrying malaria parasites in the same environment and the suitability of this novel xenosurveillancetool to support malaria transmission surveillance.

In this study, *Cx*. *quinquefasciatus* and other less frequent *Culex* species predominated in the mosquito collections. Some mosquito species, such as *Cx*. *quinquefasciatus*, have a spatial distribution and abundance, which is strongly dependent on human presence [36,40–44]. These mosquitoes are highly anthropophilic, feed frequently, and prefer to blood feed at night and often stay inside human dwellings [45–47]. *Cx*. *quinquefasciatus*, for example, are inclined to blood feed in the evening and evening twilight and they use human dwellings as shelter during the day and at night [40–42,48]. The feeding preference is for human blood and the behavior of *Cx*. *quinquefasciatus*, make this species very susceptible to pathogens present in the human host blood [6]. After the blood meal, the female mosquitoes have limited mobilityand rest on interior walls of houses for several hours, where they can be easily collected via aspiration. After capture, the blood contents present in the mosquito’s gut can be analyzed for the presence of different pathogens. Mosquitoes behave like “flying biological syringes” [36,48–52]. As these biological syringes are not competent vectors, replication is not expected [6,8,11–12,53,54] and the pathogen needs to be detected before complete digestion in the mosquito gut. During the study, we identified and separated mosquitoes by taxa to demonstrate the different possibilities for finding *Plasmodium* DNA. However, the identification is labour-intensive, though this may not be required when the proposed xenosurveillance method is used in operational settings.

A variable that needs to be taken into account in this xenosurveillance method is the sensitivity of *Plasmodium* detection in field-collected culicids. Through laser confocal microscopy, the development of *P*. *falciparum* in *Cx*. *quinquefasciatus* up to 30 hours after experimental infection was observed [55]. However, the authors point out that very few of the parasites in *Cx*. *quinquefasciatus* were alive during this period of 30 hours. The results obtained through the follow-up of *P*. *vivax* experimental feeding in *Cx*. *quinquefasciatus* demonstrated the decay of the DNA detection rates. The detection was only possible up to 48 hours after feeding. Since the mechanism of parasite killing must be sufficiently powerful that *Plasmodium* is not able to overcome it, working only with visibly engorged mosquitoes is a feasible, less costly and less laborious option, and the limit of five mosquito abdomens per pool demonstrates the operational viability of the technique. The simple cut of the abdomen of the mosquito does not require more time-consuming procedures and provides resource saving of DNA extraction and qPCR consumables, fundamental characteristics for the feasibility of any surveillance tool.

The presence of positive blood-fed culicids was significantly correlated to the force of infection and *Plasmodium* infection prevalence in humans. This correlation is important for combined collection methods and for indoor-collected culicids. In terms of logistics, using only indoor aspiration collection methods appeared to be more reliable. Although BG Sentinel traps are more productive, culicids collected by this method are mostly not engorged. BG Sentinel traps are large and difficult to carry to the field. The transportation requires vehicles with higher freight capacity and a second home visit for trap removal. Differently, the electric aspirators collect a greater proportion of engorged mosquitoes. This technique permits speed and practicality, allowing only one technician to aspire ~15 houses in one morning, in a single visit for culicid capture. Furthermore, the portability of the equipment with low requirement of logistics, allows the accomplishment of the visits even by motorcycle, which is deemed favourable in operational settings. However, in unfurnished and single-room homes, capture was usually poorly productive or even negative, which is probably due to the absence of mosquito resting sites. Considering that indoor aspiration may be considered by some as invasive, there was an expectation of rejection, which was not confirmed. Participants exhibited a good receptivity, even at times when some members of a family were still asleep.

In the force of infection analysis, the total number of blood-fed culicids was significantly associated with malaria infections. One explanation might be that some houses in poorer conditions are more permissive to *Culex* mosquitoes in parallel with higher odds to inhabitants who also have malaria. Unlike other studies, the samples of the present study were obtained in a seasonal period in which there was a lower prevalence of malaria, and in a single round of collections. Thus, the information generated may be important for estimating the circulation of the pathogen in the area, even in periods of low transmission, corroborating the possibility of using this tool as a new malaria indicator.

A good spatial correlation between *Plasmodium* DNA positive mosquitoes and infected humans was observed in the study area, where asymptomatic infection predominates. The existence of asymptomatic individuals who are carriers of *Plasmodium* sp. in significant proportions, even in a hypoendemic area, shows that the prevalence of infection, when based on the thick smear drops analyzed by microscopy, is underestimated. This indicates the importance of these reservoirs in the dynamics of malaria transmission, and suggests that the submicroscopic parasite may be important for the transmission between the high and low transmission seasonal periods [56,57]. Thus, alternatives to conventional surveillance and control measures should be implemented. In this context, the xenosurveillance method is a valuable complement to the official surveillance system.

New malaria infection burden surveillance strategies should be simple to implement, technologically uncomplicated, cost-effective and applicable to areas where malaria cases are known to occur. In this study, we found a significant association between blood-fed and *Plasmodium* DNA positive culicids and malaria prevalence. Our results suggest that xenosurveillance can be used in endemic tropical regions in order to estimate malaria burden and identify transmission foci in areas of *a P*. *vivax* asymptomatic carriers. As a perspective, this tool can be applied for the simultaneous surveillance of human pathogens, such as vector-borne, transmission areas overlapping with malaria, while taking advantage of the same collection efforts.

## Supporting information

S1 Checklist STROBE Checklist. (DOC)

S1 Dataset Study database. (XLS)
